# Outcome of patients on second line antiretroviral therapy under programmatic condition in India

**DOI:** 10.1186/s12879-015-1270-8

**Published:** 2015-11-14

**Authors:** Jaya Chakravarty, Shyam Sundar, Ankita Chourasia, Pallav Narayan Singh, Swarali Kurle, Srikanth P. Tripathy, Devidas N Chaturbhuj, Madhukar Rai, Amit Kumar Agarwal, Rabindra Nath Mishra, Ramesh S. Paranjape

**Affiliations:** Department of Medicine, Institute of Medical Sciences, Banaras Hindu University, Varanasi, 221005 India; Indian Council of Medical Research, National AIDS Research Institute, Bhosari, Pune, India; Indian Council of Medical Research, National JALMA Institute of Leprosy and Other Mycobacterial Diseases, Agra, India; Department of Community Medicine, Institute of Medical Sciences, Banaras Hindu University, Varanasi, India

**Keywords:** HIV/AIDS, Antiretroviral therapy, Virological response

## Abstract

**Background:**

The National AIDS Control Organization of India has been providing free second line antiretroviral therapy (ART) since 2008. This observational study reports the survival and virologic suppression of patients on second-line ART under programmatic condition and type of mutations acquired by those failing therapy.

**Methods:**

170 patients initiated on second-line therapy between 2008 and 2012 were followed up till 2013. Viral Load (VL) was repeated at 6 months for all patients and at 12 months for those with VL >400 copies/ml at 6 months. Adequate virological response was defined as plasma HIV-1 VL <400 copies/ml and virological failure was defined as VL >1000 copies/ml. Genotyping was done in 16 patients with virological failure.

**Results:**

Out of 170 patients, 110 (64.7 %) were alive and on therapy and 35 (20.5 %) expired. In the first year the occurrence of death was 13.7 /100 person years while between1 and 5 year it was 3.88 /100 person years. In the first year, duration of immunological failure >12 months, weight <45 kg, WHO clinical stage 3 and 4 and WHO criteria CD4 count less than pretherapy baseline [hazard ratio HR 4.2. 15.8, 11.9 & 4.1 respectively] and beyond first year poor first and second line adherence and first line CD4 count < 200/μL [HR 5.2,15.8, 3.3 respectively] had high risk of death.

119/152 (78.2 %) had adequate virological response and 27/152 (17.7 %) had virological failure. High viral load at baseline and poor second line adherence (Odds Ratio 3.4 & 2.8 respectively) had increased risk of virological failure. Among those genotyped, 50 % had major Protease Inhibitor mutation (M46I commonest) however 87.5 % were still susceptible to darunavir.

**Conclusions:**

Second line therapy has shown high early mortality but good virological suppression under programmatic conditions.

## Background

Of the 4.8 million people living with HIV (PLHIV) in Asia, nearly half (49 %) are in India. There has been a massive scale-up of antiretroviral therapy (ART) services in India since the National AIDS Control Organization (NACO) launched the ART centres providing free antiretroviral drugs in 2004. As of September 2014, there were 453 fully functional ART centres across the country providing free first line ART to 810,339 PLHIVs [1]. With the increase in number of patients on first line therapy it is envisioned that a proportion of patients will experience treatment failure and need second line ART regimens over time. In 2008, NACO piloted a national strategy for the provision of free second-line ART in India and these drugs are being provided at 10 Centres of Excellence and 16 ART Plus Centre. As on September 2014, 10,223 patients were receiving second line drugs in the National program [1].

Similar to other resource-limited settings (RLS), access to routine viral load testing to monitor therapy or genotyping before start of second line is not available in the program. Patients suspected of treatment failure on first line regimens based on WHO defined immunologic or clinical criteria are subjected to viral load testing, and only those with definitive virologic failure qualify for switch to second-line ART. Like other RLS the only second line ART available consists of a boosted protease inhibitor (bPI) with two nucleoside reverse transcriptase inhibitors (NRTIs).

Amongst the RLS, reports of efficacy and survival of patients on second line regimens are available mostly from Africa [2–4]. Although the second line ART program has been launched by NACO for six years studies to evaluate the survival and efficacy of the prevailing second line regimen are lacking in the country. A recent study from India has shown an excellent early outcome of second line treatment [5]. In the near future, NACO plans to consolidate the first and second line treatment and launch third line ART [6]. Therefore, there is an urgent need to evaluate the efficacy of the second line therapy and to know the mutations acquired by those failing therapy for the selection of an appropriate third line regimen. This study was done to report the survival and virologic suppression and their predictors in patients receiving Protease Inhibitor (PI) based second-line ART under programmatic condition and to assess the mutations acquired by those failing second line therapy.

## Methods

### Study site

This observational study was conducted at the ART Centre of the Centre of Excellence (COE), of Banaras Hindu University (BHU). The ART centre is one of the largest in this region with 18,746 PLHIV registered in HIV care and 4530 currently on first line ART. It is also a referral centre for evaluation of patients suspected of first line failure from 14 ART centres.

### Study population

The study was approved by the Ethics Committee of the Institute of Medical Sciences, Banaras Hindu University. Data of all patients >15 years of age who were started on second line therapy due to failure of first line ART at COE BHU since the beginning of the program in December 2008 to December 2012 were included in this study. Written informed consent from patients or their guardians for children were taken for HIV drug resistance genotyping.

The first line regimen recommended by NACO and received by patients was Zidovudine + Lamivudine + Nevirapine if hemoglobin was >9gm/dl or Stavudine + Lamivudine + Nevirapine if hemoglobin <9gm/dl. Efavirenz was substituted for Nevirapine in those taking antitubercular drugs and those with Nevirapine toxicity. Patients on first line therapy were eligible for evaluation for second-line ART if they had been receiving ART for at least 6 months, and had demonstrated treatment adherence of >95 %, and had WHO clinical or immunological failure as per NACO guidelines. Viral load estimation was done in these patients and those with HIV RNA >10,000 copies/mL were considered as first line failure and started on second line therapy [7]. In May 2011, the cut off level of viral load for starting ART was reduced to >5000 copies/ml by NACO [8].

All patients with first line failure between 2008 and April 2011 were given a uniform second-line ART regimen provided by NACO comprising of tenofovir (TDF) + lamivudine (3TC) + zidovudine (AZT) + lopinavir/ritonavir (LPV/r). Zidovudine was excluded from the regimen in patients with significant anemia (hemoglobin less than 9 g/dL) or previous history of zidovudine induced anemia. After May 2011, the second line ART provided by NACO was tenofovir with lamivudine and Atazanavir/ritonavir (ATV/r).All new patients and those who were previously on lopinavir based regimen were shifted to this regimen [8]. In patients with concomitant tuberculosis rifampicin was replaced by rifabutin without any change in the ART regimen.

Patients were followed up monthly and CD4 count (FACS Calibur Becton Dickinson Biosciences) was done 6 monthly for all patient. During each visit, patients were counselled for adherence and evaluated for drug toxicity, clinical improvement and opportunistic infections. Patient’s weight, clinical stage, functional status, drug toxicity, adherence to ART medication, presence of opportunistic infection, any change in therapy were documented. Viral load (VL) was repeated by COBAS TaqMan HIV-1 assay in all patients at 6 months, if it was <400copies/ml at 6 months it was not repeated further as per National guidelines. Patients with VL >400 copies/ml at 6 months, adherence was reinforced and VL was repeated at 12 months. Adherence was calculated on the basis of pill count at every visit by the formula: Number of pills actually taken by a patient for a particular time period/Number of pills prescribed for this time period × 100. For analysis we compared those with >95 % adherence at every visit with those with <95 % at any visit. At the end of the month patients were labelled as ‘on treatment’ if they picked up their drugs, ‘missed’ if they did not pick up drugs for the month, dead if they expired and ‘transferred out’ if they were transferred out to another ART centre. Those patients who did not come for 3 consecutive months were labelled as lost to follow up (LFU) at the end of fourth month as per NACO guidelines.

#### HIV drug resistance (HIVDR) genotyping

The HIVDR genotyping was performed in patients with viral load >1000 copies/ml on two occasions after 6 months of second line therapy. Plasma samples stored at −70 °C were used for detection of HIVDR mutations by in-house assay described in a previous study [9]. RNA extraction from plasma samples were performed using the NucliSENSEasyMAGTM (Biomerieux, Durham, NC) automated nucleic acid extraction system according to the manufacturer’s recommendations (NucliSENSEasyMAG user manual, v1.1; BioMe’rieux, Boxtel, Netherlands). The RobusT one step RT-PCR kit (FinnzymesOy, Finland) was used to amplify the complete protease and RT region of the *pol* gene. RT-PCR was performed as described previously using *2021 F* and *4521R* primers [10]. The nested PCR was performed using the inner primer pair*2135F* and *3338R* to get an amplified fragment of 2336 bp. A Gene Amp PCR system 9700 thermal cycler (Applied Biosystem, CA, USA) was used for all PCRs. DNA sequencing was performed on 3100 DNA genetic analyzer (Applied Biosystem, CA, USA) using a set of six specific primers (*2135 F, 2493 F, 3012 F, 2557R, 3117R, 3338R*). The raw sequence data from ABI 3100 genetic analyzer was assembled, aligned and edited with the SeqScape v2.0 software (Applied Biosystem, CA, USA). The quality of the sequences was assessed using the SQUAT software. The HIVDR mutational analysis was performed using the online “HIVdb Program: Sequence Analysis” program from Stanford University [11].

Genotype sensitivity scores (GSS) for the prescribed regimen at the time of second line failure were calculated based on the five Stanford HIVdb resistance categories: susceptible or complete activity, potential low-level resistance or good activity, low-level resistance or partial activity, intermediate-level resistance or scarce activity, high-level resistance or no activity corresponding with scores of 1.00, 0.75, 0.50, 0.25, and 0.00, respectively [12, 13]. Etravirine susceptibility scores (ESS) were calculated using the scoring system described by Vingerhoets et al. [14] and recommended by the IAS-USA.

### Statistical analysis

Data was extracted and was analysed using SPSS version 16.0. The data was presented as mean ± standard deviation (SD) for continuous variables and frequency with their respective percentages for categorical variables. Patient characteristics was described in terms of median with their inter quartile range (IQR) for skewed continuous data. For categorical data Chi-square test and Fischer Exact test was used and for continuous data Student’s *t* test and Mann Whitney *U* test was used.

For efficacy analysis, virological response at one year of therapy was observed. Adequate virological response was defined as plasma HIV-1 VL <400 copies/ml, VL between 400 and 1000 copies /ml was defined as on-going viremia and VL >1000 copies/ml was used to define virological failure or treatment failure at one year. Efficacy data was analyzed by an intention-to-treat (ITT) i.e. among all patients included in the study and on-treatment (OT) approach i.e. among those patients who at least had a VL at six month. VL at six month was included for analysis for those patients eligible for twelve month viral load but it could not be done due to death or LFU and for those who had adequate virological suppression at 6 months.

To assess the predictors of treatment failure patients with VL >1000 copies/ml were compared with those with VL < 400copies/ml. To define predictors of treatment failure, univariate analyses were performed with the following determinants: age, gender, first-line ART regimen (zidovudine versus stavudine), weight, CD4 count, clinical stage at the start of first and second-line treatment, presence of tuberculosis, adherence, duration of treatment of first and second-line therapy. A multivariate analysis was performed by using the binary logistic regression model, including all variables that were associated with the outcome (*P* < 0.05) in univariate analysis. To compare the efficacy of LPV/r and ATV/r based second line regimen, virological suppression was assessed at one year by comparing patients started on LPV/r based second line treatment between 2008 and April 2010 and a VL report before change of therapy to ATV/r with those newly started on ATV/r based regimen after May 2011with 6 or 12 month VL.

In survival analysis, patients started on second line therapy between 2008 and 2012 were included. Status of these patient was assessed in December 2013, so that all subjects had the potential to complete a minimum of 1 year of follow-up. The main outcome variable was death and the time of its occurrence during the follow up period. The data was treated as censored when either patients were lost-to follow up or transferred-out to other ART centres. The predictor variables used in the analysis were duration of immunological failure, WHO criteria for immunological failure, weight, WHO clinical stage, CD4 count, presence of tuberculosis, adherence at the start of both first and second line treatment and age, sex, viral load at the beginning of second line treatment. Variables that were statistically significant (p value <0.05) in the bivariate analysis were subsequently considered for multivariate analysis (Cox proportional hazard model) to assess the relationship between these variables and mortality.

## Results

Two hundred two patients were started on second line ART between December 2008 and December 2012, out of which 7 were already on therapy from outside and 25 were transferred out to other centre before six month viral load thus only 170 patients were included in the analysis.

Baseline characteristics of patient started on second line therapy are given in Table 1. 82.9 % were males, median duration of second line therapy was 22.50 months (IQR-14-40.25). 33 (19.4 %) patients had tuberculosis during second line treatment and adherence during second line was > 95 % in 132 (77.6 %) patients. All patients received TDF and 3TC as a part of second line regimen. Among PI, 12 patients received only LPV/r (as they had expired before change to ATV/r), 79 received both LPV/r and ATV/r and 79 received only ATV/r based PI regimen. Median CD4 count at the start of second line therapy was 78.50/μL (IQR 49.75–121.25), at 12 months 273/μL (IQR 182–357), at 24 month 319/μL (IQR 221–452), at 36 month 315/μL (IQR 209–467), at 48 month 285/μL (IQR 242–473) and at 60 month 343 /μL (IQR 228–504).

**Table 1:** Baseline characteristics of patients on second line (2^nd^ Line) ART

Variable		Number (*n* = 170)
Age(years)	Mean ± SD	36.78 ± 7.30
	Median (IQR)	35.0 (32.0-42.0)
Sex	Male n (%)	141 (82.9)
	Female n (%)	29 (17.1)
Duration of Immunological failure (months)	Mean ± SD	17.30 ± 15.71
	Median (IQR)^a^	12 (6.0-23.0)
Weight(Kg) at the start of 2^nd^ line ART	Mean ± SD	48.34 ± 9.07
	Median (IQR)^a^	49.0 (41–55)
Clinical Stage at the start of 2^nd^ line ART	I + II n (%)	118 (69.4)
	III + IV n (%)	52 (30.6)
CD4 count at the start of 2^nd^ line ART (/μL)	Mean ± SD	94.36 ± 72.98
	Median (IQR)^a^	78.50 (49.75-121.25)
Viralload baseline (copies/ml)	Mean ± SD	490000.00 ± 1150000.00
	Median (IQR)^a^	1770000 (67375–3540000)
Last NRTI regimen	Stavudine n (%)	81 (47.6)
	Zidovudine n (%)	89 (52.4)
Total Duration of 2^nd^ line ART (months)	Mean ± SD	26.65 ± 16.76
	Median (IQR)^a^	22.50 (14–40.25)
Tuberculosis during 1^st^ line ART	Yes n (%)	89 (52.4)
	No n (%)	81 (47.6)
Tuberculosis during 2^nd^ line ART	Yes n (%)	33 (19.4)
	No n (%)	137 (80.6)
1^st^ line ART Adherence	Adherence (<80-95 % ) n (%)	46 (27.1)
	Adherence (>95 %) n (%)	124 (72.9)
2^nd^ line ART Adherence	Adherence (<80 %-95 %) n (%)	38 (22.4)
	Adherence (>95 %) n (%)	132 (77.6)

^a^Inter quartile range

### Survival

At the end of follow up, out of the 170 patients started on second line therapy, 35 (20.6 %) had expired, 5 (2.9 %) were lost to follow up, 20 (11.8 %) were transferred out to other ART centres and 110 (64.7 %) were alive and on ART.

Out of the 35 patients who died, 21 (60 %) expired within 12 months of starting second line. Among them 10 had tuberculosis, one each had pneumonia, oesophageal candidiasis and diarrhea and no cause was documented in 8 patients. Among these 21 patients, viral load at 6 month was not done in 17 patients due to death, 1 had virological failure, 2 patients had adequate virological suppression and 1 had ongoing viremia.

14 patients expired after 1 year of second line. Among them 2 had tuberculosis, one each had anal carcinoma, carcinoma penis, chronic renal failure and HIV encephalopathy. Among these 14 patients 6 had virological failure, 1 had ongoing viremia, rest had adequate virological suppression. The risk of death in the first year of therapy was 13.7/100 person years while between 1 and 5 year it was 3.88/100 person years.

In the first year duration of immunological failure >12 months [HR 4.218; 95 % CI: 1.146-15.519], presence of WHO immunological criteria CD4 count less than pretherapy (at the start of first line) baseline [HR 4.111; 95 % CI: 1.050-16.096]; weight <45 kg [HR 15.777; 95 % CI: 1.734 -143.535],WHO clinical stage 3 and 4 [HR 11.871; 95 % CI: 2.695-52.296] had higher risk of death. Beyond first year, patients with CD4 count less than pretherapy baseline [HR 3.328; 95 % CI: 1.031-10.742], poor adherence during first line [HR 5.226; 95 % CI: 1.587-17.210] as well as during second line therapy [HR 15.838; 95 % CI: 4.274-58.685] had higher risk of death (Table 2).

**Table 2:** Univariate and multivariate analysis of survived and expired patients within 1 year and beyond 1 year

	Within 1 year				1-5 year			
Characteristics	Uni-variate	*p*- value	Multi-variate	*p*- value	Uni-variate	*p*-value	Multi-variate	*p*-value
	HR (95 % CI)		HR (95 % CI)		HR (95 % CI)		HR (95 % CI)	
Age (years)								
>40	1.718 (0.712-4.145)	0.229			2.508 (0.879-7.154)	0.086		
≤40	1				1			
Sex								
Male	2.059 (0.480-8.839)	0.331			0.508 (0.158-1.630)	0.255		
Female	1				1			
WHO Stage 1^st^ line								
III + IV	2.616 (1.111-6.162)	0.028	0.710 (0.202-2.499)	0.594	1.956 (0.677-5.652)	0.215		
I + II	1		1		1			
Tuberculosis 1^st^ line								
Yes	1.218 (0.513-2.891)	0.654			1.001 (0.346-2.894)	0.998		
No	1				1			
Adherence 1^st^ line								
<95 %	1.115 (0.433-2.874)	0.822			5.673 (1.778-18.094)	0.003	5.226 (1.587-17.210)	0.007
>95 %	1				1		1	
Weight (Kg) 1^st^ line								
≤45	0.867 (0.350-2.149)	0.759			2.407 (0.826-7.013)	0.107		
>45	1				1			
CD4 count 1^st^ line (/μL)								
<200	4.989 (0.670-37.178)	0.117			28.304 (0.085-924.0)	0.259		
>200	1				1			
Duration of Immunological failure (months)								
>12	3.262 (1.182-11.123)	0.024	4.218 (1.146-15.519)	0.030	2.684 (0.824-8.745)	0.101		
< 12	1		1		1			
Clinical failure								
Yes	2.721 (1.098-6.743)	0.031	1.056 (0.240-4.645)	0.943	0.640 (0.143-2.869)	0.560)		
No	1		1		1			
CD4 count less than baseline								
Yes	3.579 (1.204-10.638)	0.022	4.111 (1.050-16.096)	0.042	2.120 (0.709-6.343)	0.179	3.328 (1.031-10.742)	0.044
No	1		1		1		1	
50 % fall from peak CD4 count								
Yes	0.448 (0.174-1.154)	0.096			25.70 (0.038-1719.0)	0.328		
No	1				1			
CD4 count < 100/μL								
Yes	2.100 (0.815-5.413)	0.125			3.038 (0.847-10.895)	0.088		
No	1				1			
CD4 count 2^nd^ line (/μL)								
<200	0.644 (0.150-2.765)	0.554			22.856 (0.003-1692.0)	0.491		
>200	1				1			
WHO Stage 2^nd^ line								
III + IV	15.854 (4.666-53.872)	<0.001	11.871 (2.695-52.296)	0.001	1.446 (0.484-4.318)	0.509		
I + II	1		1		1			
Weight (Kg) 2^nd^ line								
≤45	6.491 (1.512-27.877)	0.012	15.777 (1.734-143.535)	0.014	1.089 (0.363-3.269)	0.879		
>45	1		1		1			
Viral Load 2^nd^ Line (copies/ml)								
>177000	2.042 (0.824-5.061)	0.123			0.790 (0.274-2.279)	0.662		
≤177000	1				1			
Tuberculosis 2^nd^ line								
Yes	4.219 (1.791-9.939)	0.001	2.336 (0.752-7.256)	0.142	0.854 (0.190-3.837)	0.837		
No	1		1		1			
Adherence 2^nd^ line								
<95 %	1.483 (0.575-3.821)	0.415			14.682 (4.093-52.660)	<0.001	15.838 (4.274-58.685)	<0.001
>95 %	1				1		1	

### Efficacy

At 6 months viral load was done in 152 patients as 17 had expired and 1 was LFU. 102 patients had a viral load of <400 copies/ml, 12 had viral load between 400 and 1000 copies/ml and 38 had viral load >1000 copies/ml. At 12 months viral load was repeated in only 41 patients out of the 50 patients whose VL was >400 copies/ml at six month, as 5 expired and 4 were LFU. Among these 41 patients, 17 had VL <400 copies/ml, 5 had VL between 400 and 1000 copies/ml and 19 had viral load >1000 copies/ml.

Overall, out of 170 patients who were started on second line, 119/170 i.e. 70 % by ITT and 119/152 i.e. 78.2 % by OT had adequate virological response, 6 (3.5 % ITT, 3.9 % OT) had ongoing viremia and 27 (15.8 % ITT, 17.7 % OT) had virological failure at one year. Viral load ≥177,000 copies/ml at baseline (OR 3.402, 95 % CI: 1.272-9.097) and <95 % adherence during second line treatment (OR 2.788, 95 % CI: 1.044-7.445) was significantly associated with second line failure (Table 3). On comparing the efficacy of LPV/r vs ATV/r, the two PIs used as second line, 85.2 % (46/54) had virological suppression with LPV/r while 69 % (49/71) had virological suppression with ATV/r at one year.

**Table 3:** Univariate and multivariate analysis between virological Failure and adequate virological response

Characteristics	Uni-variate	*p*-value	Multi-variate	*p*-value
	Odds ratio (95 % CI)		Odds ratio (95 % CI)	
Age (years)				
>40	1.249 (0.496-3.147)	0.637		
≤40	1			
Sex				
Male	0.423 (0.161-1.112)	0.081		
Female	1			
WHO Stage 1^st^ line				
III + IV	1.038 (0.400-2.698)	0.938		
I + II	1			
Tuberculosis 1^st^ line				
Yes	2.327 (0.968-2.327)	0.059		
No	1			
Adherence 1^st^ line				
<95 %	2.600 (1.090-6.201)	0.031	1.918 (0.741-4.962)	0.180
>95 %	1		1	
Weight (Kg) 1^st^ line				
≤45	1.215 (0.517-2.854)	0.655		
>45	1			
CD4 count 1^st^ line (/μL)				
<200	3.618 (1.455-8.998)	0.006	2.788 (1.045-7.439)	0.041
>200	1		1	
Duration of Immunological failure (months)				
>12	0.542 (0.229-1.283)	0.164		
<12	1			
Clinical failure				
Yes	5.253 (0.673-40.976)	0.114		
No	1			
CD4 count less than baseline				
Yes	2.335 (0.949-5.749)	0.065		
No	1			
50 % fall from peak CD4 count				
Yes	1.092 (0.339-3.519)	0.882		
No	1			
CD4 count < 100/μL				
Yes	0.688 (0.297-1.593)	0.382		
No	1			
CD4 count 2^nd^ line (/μL)				
<200	3.275 (0.856-12.537)	0.083		
>200	1			
WHO Stage 2^nd^ line				
III + IV	1.287 (0.477-3.477)	0.618		
I + II	1			
Weight (Kg) 2^nd^ line				
≤45	1.143 (0.492-2.653)	0.756		
>45	1			
Viral Load 2^nd^ Line (copies/ml)				
>177000	3.558 (1.399-9.050)	0.008	3.402 (1.272-9.097)	0.015
≤177000	1		1	
Tuberculosis 2^nd^ line				
Yes	1.364 (0.455-4.090)	0.580		
No	1			
Adherence 2^nd^ line				
<95 %	2.870 (1.176-7.004)	0.021	2.788 (1.044-7.445)	0.041
>95 %	1		1	

### HIVDR genotyping

Out of the 27 patients who failed second line therapy 3 expired and 2 were LFU and sample for genotyping could not be collected in 3 patients. Out of the 19 samples available, genotyping data of only 16 patients could be included in the study as sample (SL) 7, 17 and 18 did not amplify. All samples were HIV 1 virus subtype C. 50 % (8/16) had major PI mutation, 62.5 % (10/16) had minor PI mutation, 81.25 % (13/16) had NRTI mutation and 93.75 % (15/16) had NNRTI (non nucleoside reverse transcriptase) mutation (Table 4). Median GSS was 1.0 (0–1.81).

**Table 4:** Mutations acquired by patients failing second line treatment

Patient ID	Gene Bank Accession No.	Duration of second line treatment (months)	Adherence during 2^nd^ line treatment	Subtype	Major PI mutation	Minor PI mutation	NRTI mutation	NNRTI mutation	GSS score	Etravirine sensitivity score
SL-1	KJ933454	46	>95 %	C	M46I, I47A, I50IL, I84V	A71V	M41L, D67N, K70R, L74IL, M184V, T215Y, K219Q	K103N, K238KN	0	0
SL-2	KJ933455	13	>95 %	C	NONE	NONE	M41L, D67N, M184V, T215F, K219W	Y181V, G190A	1.25	4
SL-3	KJ933456	23	>95 %	C	M46I, N88S	L24I, Q58E, A71V	M41L, K65R, K70T, M184V	A98G, K101E, G190A	0	3
SL-4	KJ933457	9	>95 %	C	NONE	NONE	M184V	K101E, G190A	2	2
SL-5	KJ933458	20	>95 %	C	M46I	L23IL	D67N, V75M, M184V	Y181C	1.25	2.5
SL-6	KJ933459	24	>95 %	C	None	K20I, L90LW	None	V108IV, Y181C, H221Y	3	2.5
SL-9	KJ933461	25	<95 %	C	M46I, I50L, V82A	L33FL, A71AV, G73S, N83DN	M41L, D67N, V75M, M184V, L210W, T215Y, K219N	A98G, K103N	0	1
SL-11	KJ933463	9	>95 %	C	M46I, N88NS	K20IT, E35DEG, A71AT, T74S	M41L, M184V, L210W, T215Y, K219KN	K101H, Y181CFIS, G190A	0	7.5
SL-12	KJ933464	14	>95 %	C	NONE	K20KT, G73S	M41L, D67E, V75M, M184V, L210W, T215Y	V90I, V108I, Y181C	0.5	3.5
SL-13	KJ933465	10	>95 %	A1C	I84IV	L23I, A71V	M41L, D67N, L74I, V75M, M184V, L210W, T215Y, K219N	A98G, K103N, G190	0	2
SL-14	KJ933466	9	<95 %	C	None	None	None	None	3	0
SL-15	KJ933467	20	>95 %	C	NONE	NONE	M41L, D67N, V75M, M184V, T215Y, K219N	V90I, K103N	1.25	1
SL-16	KJ933468	20	>95 %	C	NONE	T74S	M41L, K70R, L74I, M184V, T215F, K219W	V106M, V108I, Y181C, F227L	1.25	2.5
SL-19	KJ933469	12	<95 %	C	NONE	NONE	NONE	K101E	3	1
SL-20	KJ933470	36	>95 %	C	M46I	NONE	M41L, D67N, T69D, V75M, M184V, L210W, T215Y	A98G, K101EK, V108I, V179IT, G190A, F227L	0.75	4
SL-21	KJ933471	18	<95 %	C	N88S	L10F, K20T, Q58E	D67N, T69D, K70R, M184V, T215F, K219Q	K101E, Y181C	0.25	3.5

Among PI mutations, M46I (*n* = 5) was the commonest mutation, followed by N88S (*n* = 3), I50L (*n* = 2), I84V (*n* = 2), V 82A (*n* = 1). 6 patients with >12 months of second line therapy as compared to 2 patients with <12 months therapy had at least one major PI mutation. Fourteen (87.5 %) patients were susceptible to darunavir (DRV) while two were associated with low level resistance to DRV. 6 (37.5 %) patients were associated with high resistance, 3 (18.75 %) had low level resistance and 7 (43.75 %) were still susceptible to atazanavir (ATV).

M184V was the commonest NRTI mutation and was present in 13 out of 16 patients. 62.5 % (*n* = 10) patients had ≥3 TAM (Thymidine analogue mutations). 81.25 % (*n* = 13) had more than 1 NNRTI mutation.Y181C and G190A was the most common NNRTI mutation present in 37.5 % (*n* = 6) patient each. ESS were ≥ 2.5 in 56.25 % (9/16) patients.

## Discussion

This study reports the outcome of patients receiving second line antiretroviral therapy under the National AIDS control programme of India. Retention in care at the end of follow up was similar to a study from rural South Africa where routine virological monitoring is done. While in our study mortality was higher, lost to follow up was much lesser than this cohort from South Africa [14]. Early mortality was high in our study but was comparable to a study from Malawi where WHO defined immunological and clinical failure criteria were used to detect first line failure similar to our study [2]. Risk factor for early death (within one year) in our study was duration of immunological >12 months, presence of WHO immunological criteria, CD4 count less than pretherapy, weight <45 kg, WHO clinical stage III &IV, all of which suggests that patients failing first line for a long time and those in poor clinical condition at the start of second line were at increased risk of death. Similar findings were observed in the study from Malawi where clinical failure at baseline and body mass index <18.5 were risk factors for death [2].

These finding suggests that delay in detecting first line failure may be the main reason for this high early mortality in our study. Studies have shown that WHO immunological failure criteria have low sensitivity for detecting virological failure [15, 16]. Moreover, the cut off viral load level for starting second line in our study was much higher (5000–10,000 copies/ml) as compared to other studies [2, 14]. A recent study from Africa has shown that delayed switch of antiretroviral therapy after virological failure is associated with increased mortality [17]. Decreasing the cut–off level for viral load for starting second line to 1000 copies/ml and ensuring early referral through education of medical officers are some of the initiatives taken by NACO which will decrease the mortality among second line patients in future. However, increasing the accessibility of viral load testing as recommended by the recent WHO guidelines would definitely go a long way in improving the second line program.

Efficacy analysis showed that 119 (70 % by ITT, 78.2 % OT) had adequate virological response at one year. Similar findings were observed in studies from other resource limited settings [4, 14], while a study from India showed slightly better outcome [5]. A recent large trial from Africa has shown adequate virological suppression in 86 % of patients at 96 weeks with the WHO recommended second line regimen of NRTI and boosted PI [18]. High early mortality, higher viral load cut off for starting therapy in our study could be the reasons for the difference in virological response from this study.

Poor adherence during second line therapy was an important risk factor for virological failure in our study similar to other studies [2, 3, 19]. Poor adherence was also a major risk for death beyond first year in our study. The fact that 50 % (7/14) of our patients who died after one year of therapy did not have adequate virological suppression, suggests that poor adherence might have led to virological failure and death.

Interestingly, 17/50 (34.0 %) of our patients who had ongoing viremia or virological failure at 6 months adequately suppressed their viral load at 12 months. Similar findings were observed in a study where 62 % patients suspected of second-line ART failure, responded to enhanced adherence support and had a two-log decrease in their level of HIV on subsequent VL testing [20]. These findings further endorses the WHO guidelines which recommends that patients failing virologically be subject to an adherence support intervention, after which a second viral load test should be performed prior to deciding on a regimen change. High viral load at baseline as a risk factor for virological failure was also recently observed in the results of TREAT Asia HIV observational database [21].We observed that virological suppression was better in those with LPV/r as compared to ATV/r, however, as this analysis included only a small subset of patients further studies are needed to come to any conclusions.

This is the first study to report the mutations acquired by second line failure patients in the National program. Median GSS was very low (1.0) in our study. 62.5 % patients had ≥3 TAM which they must have acquired during first line therapy and suggests that patients were failing on first line therapy long before detection. It also implies that they were relatively on PI monotherapy and at risk of developing PI mutations. 93.75 % of the patients in our study had NNRTI mutation, which may be due to re-emergence of archived mutations from first-line NNRTI regimens. Etravirine susceptibility scores (ESS) was ≥ 2.5 in 56.25 % patients, which is a concern as best virologic suppression are seen when the ESS score is less than or equal to 2.0 and this drug is also a part of the WHO recommended third line regimen. 50 % of our second line failure patients had major PI mutation which is much higher than previous studies [22, 23] but is similar to a recent study from Nigeria and a tertiary care centre in India [24, 25]. Patients with longer duration of therapy had more chances of acquiring PI mutation in our study, similar to the study from Nigeria [24]. Studies have shown that several *Gag* substrate mutations can cause drug resistance mutations that confer PI resistance in the absence of protease mutations [26–28]. This could explain the fact that only 50 % of the patients had PI mutation while all of them were failing second line. M46I was the commonest PI mutation observed in our study which reduces susceptibility to all PI except darunavir [11]. Similar findings were observed in other studies from India, [25, 29]. As M46I mutation has also been observed in few PI naïve patients we could have slightly overestimated the selection of this mutation in our study as genotyping was not performed before the start of second line therapy [30, 31]. N88S was the second most common PI mutation in our study which is associated with resistance to ATV/r and nelfinavir. Only two patients had I84V mutation which confers broad spectrum resistance to all PIs and low level resistance to DRV. Thus, most patients were still susceptible to DRV the recommended PI for third line regimen by WHO.

The major limitations of our study was the inability to do viral load testing for all patients at 12 months and the fact that genotyping was not done at the start of second line therapy.

## Conclusion

In this observational study there was high early mortality but good long term outcome as well as virological suppression in patients starting second line therapy under programmatic conditions in India. This early mortality can be circumvented by introducing routine virological monitoring in the program which will help in early detection of patients with failure. Virological response in our cohort was similar to other resource poor settings. Although 50 % of our second line failure patients had major PI mutation most were still susceptible to darunavir. Thus, darunavir in combination with integrase inhibitor which has not been used in the program remains a good option as a third line ART for the National program.

## Abbreviations

ART: antiretroviral therapy
ATV/r: atazanavir/ ritonavir
bPI: boosted Protease Inhibitors
DRV: darunavir
ESS: etravirine susceptibility scores
GSS: genotype sensitivity scores
HIVDR: human immunodeficiency virus drug resistance
LFU: lost to follow up
LPV/r: lopinavir/ritonavir
NACO: National AIDS control organization
NNRTI: nonnucleoside reverse transcriptase inhibitors
NRTI: nucleoside reverse transcriptase inhibitors
PLHIV: people living with HIV,VL-viral load
RLS: resource limited settings
TAM: thymidine analogue mutations
TR out: transfer out
